# Analytical and clinical performance evaluation of a new NT-proBNP assay

**DOI:** 10.1186/s12872-024-03994-w

**Published:** 2024-07-05

**Authors:** Pingfeng Feng, Junlong Qin, Zhixin Chai, Yajie Zhang, Minghai Zhao, Liya Liu, Lijun Zhang, Yaqiong Chen, Yufeng Xiong

**Affiliations:** 1 Department of Medical Laboratory, Nanfang Hospital Southern Medical University, No.1838, Guangzhou Avenue North, Guangzhou, 510515 China; 2 Medical Laboratory of ShenzhenLuohu Hospital Group, Shenzhen, 518005 China

**Keywords:** NT-proBNP, Performance verification, Reference range

## Abstract

**Background:**

The study evaluated the performance of the Mindray N-terminal pro-B-type natriuretic peptide (NT-proBNP) in a healthy population in China, focusing on creating a reference range for future clinical applications adjusted according to different demographics.

**Methods:**

The study measured NT-proBNP in 2277 healthy individuals. We analyzed age and sex-stratified data, performed precision, accuracy, linearitcvy, and detection limit studies, and evaluated method comparison and consistency between Roche and Mindray assays on 724 serum samples. We used Excel 2010, Medcalc, and GraphPad Prism 9.

**Results:**

In males, the 97.5th centile NT-proBNP concentration at age < 45, 45 to 54, 55 to 64, 65 to 74 and ≧ 75 were 89.4 ng/L, 126 ng/L, 206 ng/L, 386 ng/L and 522 ng/L, respectively. In females, the concentration of NT-proBNP at the same age was 132 ng/L, 229 ng/L, 262 ng/L, 297 ng/L and 807 ng/L, respectively. The repeatability precision coefficient of variation (CV%) for NT-proBNP was between 0.86 and 1.65 in analytical performance. In contrast, the reproducibility precision (CV%) for NT-proBNP was between 1.52 and 3.22, respectively. The study found a bias of accuracy of 3.73% in low-value samples (concentration: 148.69) and 7.31% in high-value samples (concentration: 1939.08). The sensitivity, specificity, positive predictive value (PPV), and negative predictive value (NPV) of 125 ng/L were 96.6%, 92.3%, 84.2%, and 98.5%, respectively. In contrast, those of 300 ng/L were 94.0%, 98.2%, 95.7% and 97.5%, respectively.

**Conclusions:**

The Mindray NT-proBNP assay showed increased levels in both males and females with age, with higher levels in women. It performs well and aligns with manufacturer specifications. We recommend adjusting cutoff values based on demographic factors.

**Supplementary Information:**

The online version contains supplementary material available at 10.1186/s12872-024-03994-w.

## Background

The B-type natriuretic peptide is a member of the natriuretic peptide family, which is thought to have evolved for the common homeostatic aim of circulatory system volume, osmosis, and pressure regulation and is mostly released by the heart. Pre-proBNP (134 amino acids) is transformed to B-type natriuretic peptide precursor proBNP (108 amino acids) under the effect of myocardial wall stress, myocardial Ischemia, increased cardiac load, and endocrine/paracrine regulation by other neurohormones and cytokines. It was subsequently broken into 32 amino acids of physiologically active BNP and 76 amino acids of biologically inactive N-terminal pro-B-type natriuretic peptide (NT-proBNP) [1–3].

Many clinical guidelines [4–6] advocate BNP and NT-proBNP in managing heart failure, including screening, diagnosis, severity, and prognostic assessment of heart failure (HF). The AHA/ACC/HFSA Guideline [4] recommends NT-proBNP as a screening test in patients at risk of developing HF. Increasing NT-proBNP levels (> 125 ng/L) indicate increasing filling pressures and should be followed by team-based therapy to avoid the development of cardiac dysfunction or new-onset HF. The European Society of Cardiology [5] supports NT-proBNP as a differential diagnostic test in individuals suspected of having heart failure.

In non-acute and acute situations, cutoffs of 125 and 300 ng/L are used to rule out HF in the presence of symptoms and/or signs of HF. In the acute context, 450 ng/L, 900 ng/L, and 1800 ng/L are suggested for HF, presumably in people aged 50, 50 to 75, and > 75, respectively. Although clinically applicable cutoff values for NT-proBNP have been extensively advocated and a consensus for clinical use has been formed, further evidence is needed about concentration distribution and factors impacting the biomarker in persons without known cardiovascular disease. NT-proBNP is a simple marker that can be used to assess the severity and prognosis of heart failure. Its level and change could aid in discussing prognosis and optimizing Guideline-directed medical therapy (GDMT) [6]. This study aimed to include influencing elements such as gender and age groups to provide the NT-proBNP reference value for each group.

Furthermore, analytical performance is critical for clinical applications. NT-proBNP has a prolonged half-life (about 120 min) and high storage stability [7, 8]. The results are comparable because most NT-proBNP tests use the same antibodies and calibrators as the Roche assay. The performance of the novel Mindray NT-proBNP assay was tested on the Mindray CL-6000i (Nanfang Hospital, Guangzhou, China) in this study, and a methodological comparison with the Cobas e601 was performed at the same site.

## Methods

### Ethics

The study was approved by the Medical Ethics Committee of Nanfang Hospital Southern Medical University (NFEC-2023-168). Informed consent was obtained from all subjects.

### Specimens

Fresh serum samples from healthy individuals during physical examination in Nanfang Hospital in China from April to October 2022.

### Reagents and instruments

Reference range establishment and performance verification of NT-proBNP were conducted on Mindray CL-6000i, and Mindray N-terminal pro-B-type natriuretic peptide (NT-proBNP) kit and its associated calibrators, quality control products and consumables were used. The batch number of NT-proBNP, NT-proBNP calibration, low-value control (L) and high-value control (H) was 2,022,030,500. Maintain, calibrate and perform daily quality control of CL-6000i based on the manufacturer’s requirements, establish the reference range and verify the performance of NT-proBNP under the condition that the quality control is under control.

The main components of the kit include mouse anti- NT-proBNP antibody, sheep anti-NT-probNP antibody-alkaline phosphatase marker. The Limit of Blank (LOB), Limit of Detection (LOD) and Limit of Quantitation (LOQ) are 6 ng/L, 10ng/L and 30 ng/L, respectively. The acceptable total error ≤ 30%. The measure range is 10 ng/L to 35,000 ng/L. Values below the Limit of Detection (LOD) are reported as < 10 ng/L. Values above the measuring range are reported as > 35,000 ng/L (the upper limit can be extended to 350,000 ng/L after 10-fold dilution).

### Reference range establishment

#### Participants

Healthy adults who underwent physical examinations from April to October 2022 were selected. 2277 individuals met the inclusion criteria, including 1332 males, of which 610 are aged < 45 years old, 310 are aged 45–54 years old, 151 are aged 55–64 years old, 140 are aged 65–74 years old, and 121 are aged ≥ 75 years old; and 945 females, of which 437 are aged < 45 years old, 139 are aged 45–54 years old, 122 are aged 55–64 years old, 123 are aged 65–74 years old, and 124 are aged ≥ 75 years old.

#### Inclusion criteria

There were no recent heart attacks or usage of heart-related drugs, and no history of respiratory or kidney problems. The electrocardiography (ECG), echocardiogram, glucose, lipid, liver function, and HbA1c tests are normal. Serum creatinine levels were normal, and the glomerular filtration rate (eGFR) was more than 60 mL/min/1.73 m^2^.

### Performance verification

#### Precision

Refer to American Clinical and Laboratory Standards Institute (CLSI) EP15-A2 [9], quality control L and H supplied by the manufacturer, and human NT-proBNP serum sample concentrations of 125ng/L, 300ng/L, 450ng/L, 600ng/L, 900ng/L, and 1800ng/L were analyzed. Each sample was tested continuously 20 times in one day, and the repeatability precision coefficient of variation (CV) was computed. For five days, each sample was analyzed three times every day. The reproducibility precision CV was computed. The CV of repeatable and repeatability accuracy must be less than 1/4 TEa and 1/3 TEa, as defined by the Revised Regulations for Clinical Laboratory Improvement (CLIA ‘88). Repeatable CV and repeatability precision must be within 3.25% and 4.33%, respectively.

#### Accuracy

Validate the accuracy of the manufacturer’s traceable samples at two levels, as specified in the CLSI EP15-A3 document [10]. For five days, the test was repeated twice a day. Examine the bias between the mean value of the data and the concentration goal values provided. The relative bias (mean value of the outcomes minus concentration goal value) divided by concentration target value times 100%. The relative bias is within the 1/2TEa allowed by CLIA ‘88; 12.5% is considered to have passed verification.

#### Linearity

According to the American Committee for Clinical Laboratory Standardisation (NCCLS) document EP6-A3 [11], a high-value serum sample (H) close to the manufacturer’s upper limit of linearity range (concentration 40,000 ± 5000ng/L) and a low-value serum sample (L) close to the lower limit (concentration 10ng/L) were obtained. To generate six concentration samples to test, H and L samples were combined in the following order: 1 H, 4/5H + 1/5L, 3/5H + 2/5L, 2/5H + 3/5L, 1/5H + 4/5L, and 1 L. Each was tested three times, with the average value determined. Based on the measured mean and theoretical values, the linear regression equation was created, and the linear correlation coefficient R was obtained, with ≧ 0.9900 being regarded as satisfactory.

#### Detection limit

CLSI EP17-A2 [12] is referred to. Three calibrator C0 samples from the manufacturer were chosen as LOB test samples, three serum samples with concentrations ranging from 6 to 10 ng/L as LOD test samples, and three serum samples with concentrations ranging from 10 to 30 ng/L as LOQ test samples. Each sample was tested twice daily for four days in a row, yielding 24 test results for LOB, LOD, and LOQ. The LOB and LOD test result is acceptable if no more than three findings are greater than the manufacturer standard (LOB = 6 ng/L, LOD = 10ng/L, LOQ = 30 ng/L, The deviation of LOQ for all results is less than 30%). The LOQ test yields a satisfactory result if the total error of three outcomes is less than 30% (Total error = bias + 2*CV).

#### Method comparison

Refer to NCCLS document EP9-A2 [13] to collect fresh serum samples from different patients. Sample concentrations should be evenly distributed within the detection range of the reagent and cover all clinically valuable cutoff values of NT-proBNP. Testing samples in Roche Cobas 601 Electrochemical Luminescence automatic immunoassay system and with Elecsys proBNP II reagent as the reference system and reagent, Mindray CL-6000i automatic chemiluminescence immunoassay analyzer and N-terminal pro-B-type natriuretic peptide (NT-proBNP) chemiluminescence assay reagent as the instrument and reagent to be evaluated. Every sample was measured on the two systems. Test results were used to calculate the linear regression equation, in which the results of the reference system were the X axis, and the results were evaluated as the Y axis. Correlation coefficient R was obtained, and if R^2^ was no less than 0.950, the two systems were considered to be in good agreement. Using 125ng/L and 300ng/L to evaluate the coincidence rate of the two system, the acceptable outcome is the rate of more than 90%.

#### Diagnostic performance

Patients diagnosed with congestive heart failure (CHF), which is failure to meet the systemic demands of circulation caused mainly by coronary artery disease and diabetes mellitus [14], were selected from April to October 2022. Fresh serum samples from 970 patients confirmed to be diagnosed with CHF were tested, and diagnostic performance of the medical decision levels was analyzed by comparing with the results in healthy adults in the same age groups (Supplementary Table 1).

### Statistical analysis

Excel 2010, Medcalc, and GraphPad Prism 9 statistical tools were utilized for data analysis and processing. To compare and analyze normally distributed data, the T-test was utilized. Mann-Whitney test was utilised in non-normally distributed data analysis, and metrological data were displayed as medians and percentiles. For consistency analysis, linear regression was utilised.

## Results

### Serum NT-proBNP distribution in healthy adults of different ages and genders

**Fig. 1:**
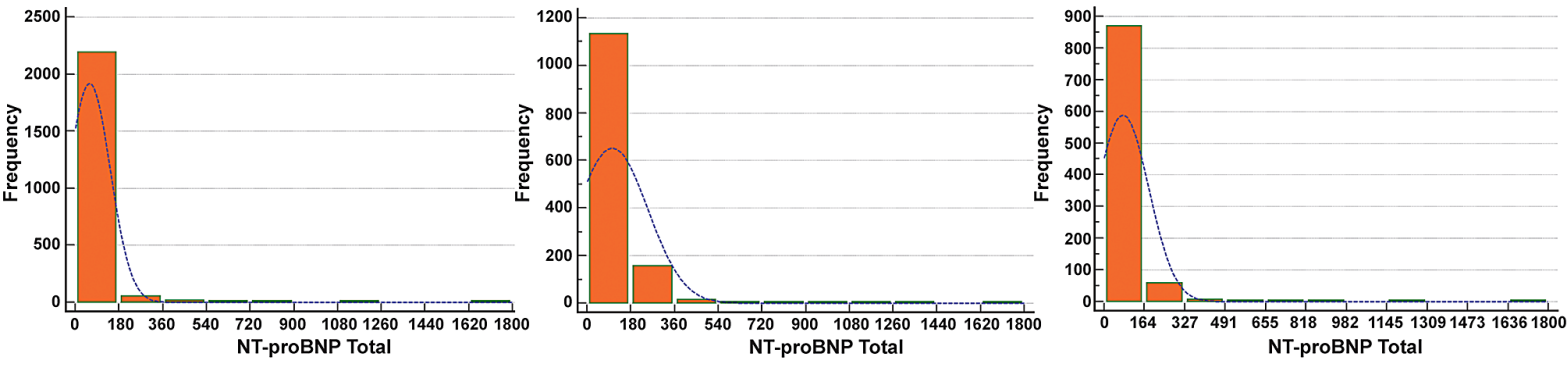
Data distribution of reference sample. **A:** Data distribution of all samples, **B:** Data distribution of male samples, C: Data distribution of female samples

Of the 2277 participants, 2206 serum samples were collected and analyzed by K-S test for normal distribution (exclusion of 71 samples with a lower concentration than the measurement range (10-35000 ng/L)). All groups (of different genders and ages) rejected normality distribution (*P* < 0.0001) **(**Fig. 1**)**.

### Analysis of NT-proBNP levels with grouping

**Fig. 2:**
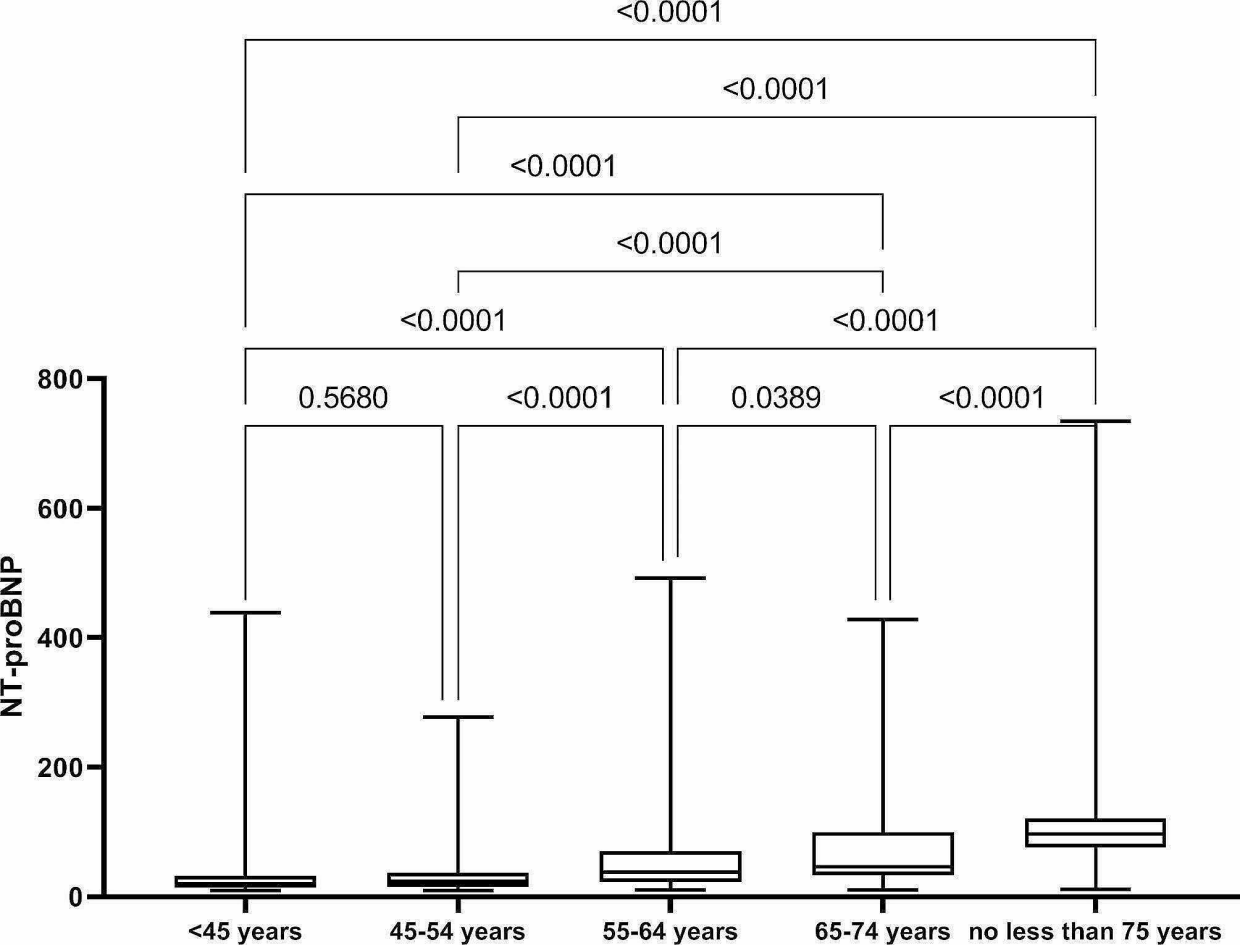
Analysis result of group difference in male

**Fig. 3:**
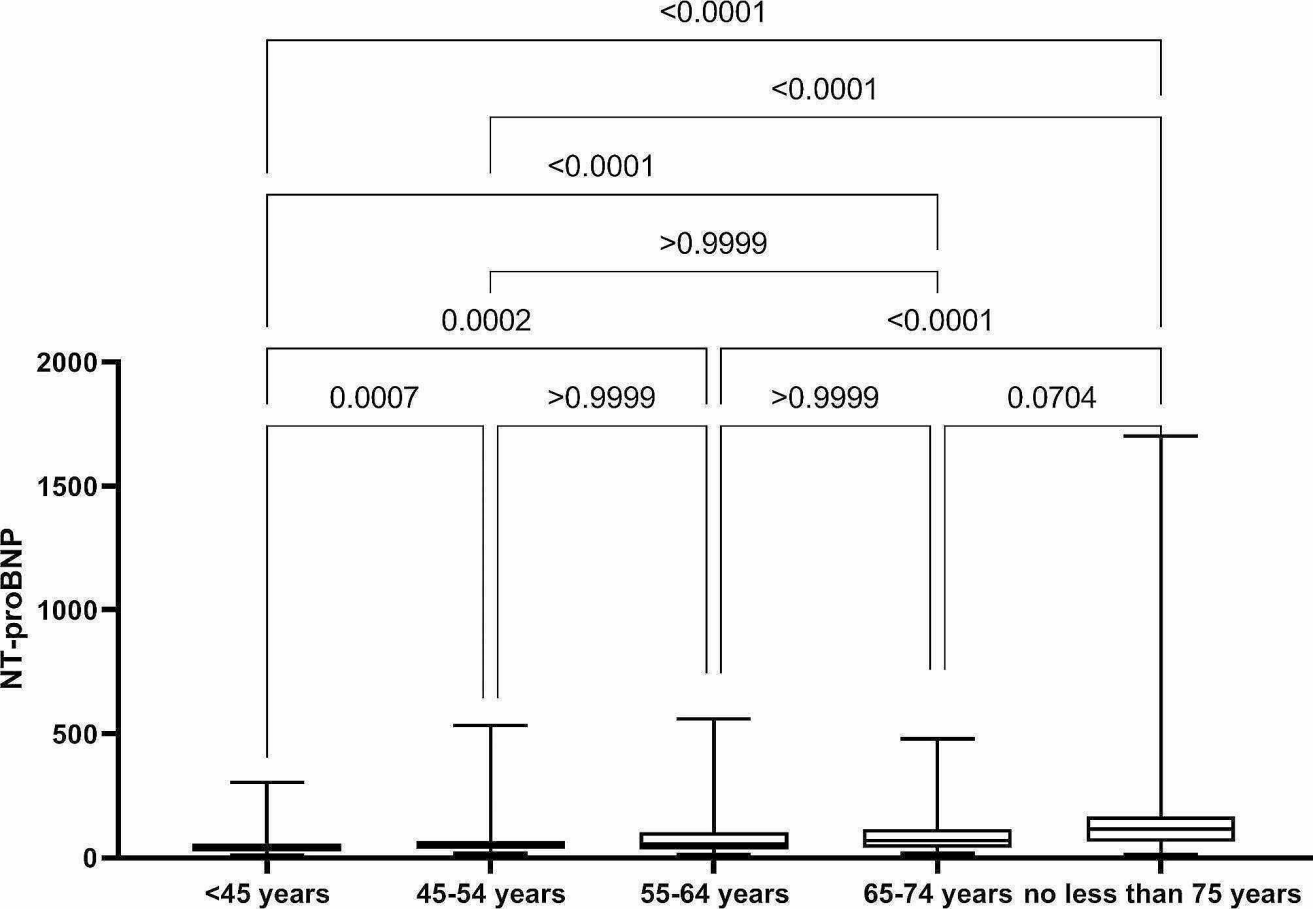
Analysis result of group difference in female

Participants were divided into groups according to the above age and gender. We performed an Unpaired t-test (between males and females) and a Kruskal-Wallis analysis (among different age groups in males and females, respectively). The results show that the *P* value < 0.0001 in males vs. females and different age subgroups in each sex **(**Figs. 2 and 3**)**. The difference between groups was statistically significant. Therefore, the reference range of NT-proBNP in healthy individuals was considered to be presented by gender and age.

### Reference range of NT-proBNP in healthy adults

Based on gender and age (< 45 years old, 45–54 years old, 55–64 years old, 65–74 years old, and ≧ 75 years old), NT-proBNP data were grouped and statistically analyzed. Table 1 shows NT-proBNP concentrations were higher in females than males and an increase in NT-proBNP with age in both sexes.

**Table 1:** Descriptive statistical analysis of NT-proBNP concentration

Total						
Age (Years)	18–44	45–54	55–64	65–74	≧ 75	Total
Number	1047	449	273	263	245	2277
Median (ng/L)	26.1	30.2	43.3	53.2	102	35.2
95th percentile (ng/L)	93.0	117	189	277	504	165
97.5th percentile (ng/L)	110	166	258	355	726	245
Male						
Age (Years)	18–44	45–54	55–64	65–74	75 or above	Total
Number	610	310	151	140	121	1332
Median (ng/L)	19.0	22.5	35.6	46.5	96.4	25.9
95th percentile (ng/L)	63.2	83.7	158	248	490	125
97.5th percentile (ng/L)	89.4	126	206	386	522	187
Female						
Age (Years)	18–44	45–54	55–64	65–74	75 or above	Total
Number	437	139	122	123	124	945
Median (ng/L)	36.0	52.5	54.3	69.4	114	48.3
95th percentile (ng/L)	108	165	241	277	645	186
97.5th percentile (ng/L)	132	229	262	297	807	281

### Precision

The CV of repeatability and reproducibility precision in quality controls and serum samples covering clinically valuable cutoff values were 0.86 -1.65% and 1.52 -2.96%, respectively (Inaccuracy (repeatability) CV ≤ 5%, indoor precision CV ≤ 10%). All meet the 1/4 TEa and 1/3 TEa requirements of NT-proBNP in CLIA ‘88 **(**Table 2**)**.

**Table 2:** Precision verification of NT-proBNP

	QCL	QCH	Sample 1	Sample 2	Sample 3	Sample 4	Sample 5	Sample 6
Repeatability(CV)	1.60%	0.90%	1.55%	1.11%	1.18%	0.86%	1.65%	1.15%
Reproducibility(CV)	2.96%	2.52%	3.22%	2.81%	2.34%	2.88%	1.52%	2.11%

QCL, low concentration of quality control level; QCH, high concentration of quality control level

### Accuracy

The deviation range of accuracy samples (low and high values) was 1.44 -7.31%, which conforms to 1/2 TEa approved by CLIA ‘88 **(**Table 3**)**.

**Table 3:** Accuracy verification of NT-proBNP

	Mean (ng/L)	CV (%)	Nominal concentration (ng/L)	Bias
Low-value sample	154.23	1.98%	148.69	3.73%
High-value sample	2100.31	1.44%	1939.08	7.31%

### Linearity

**Fig. 4:**
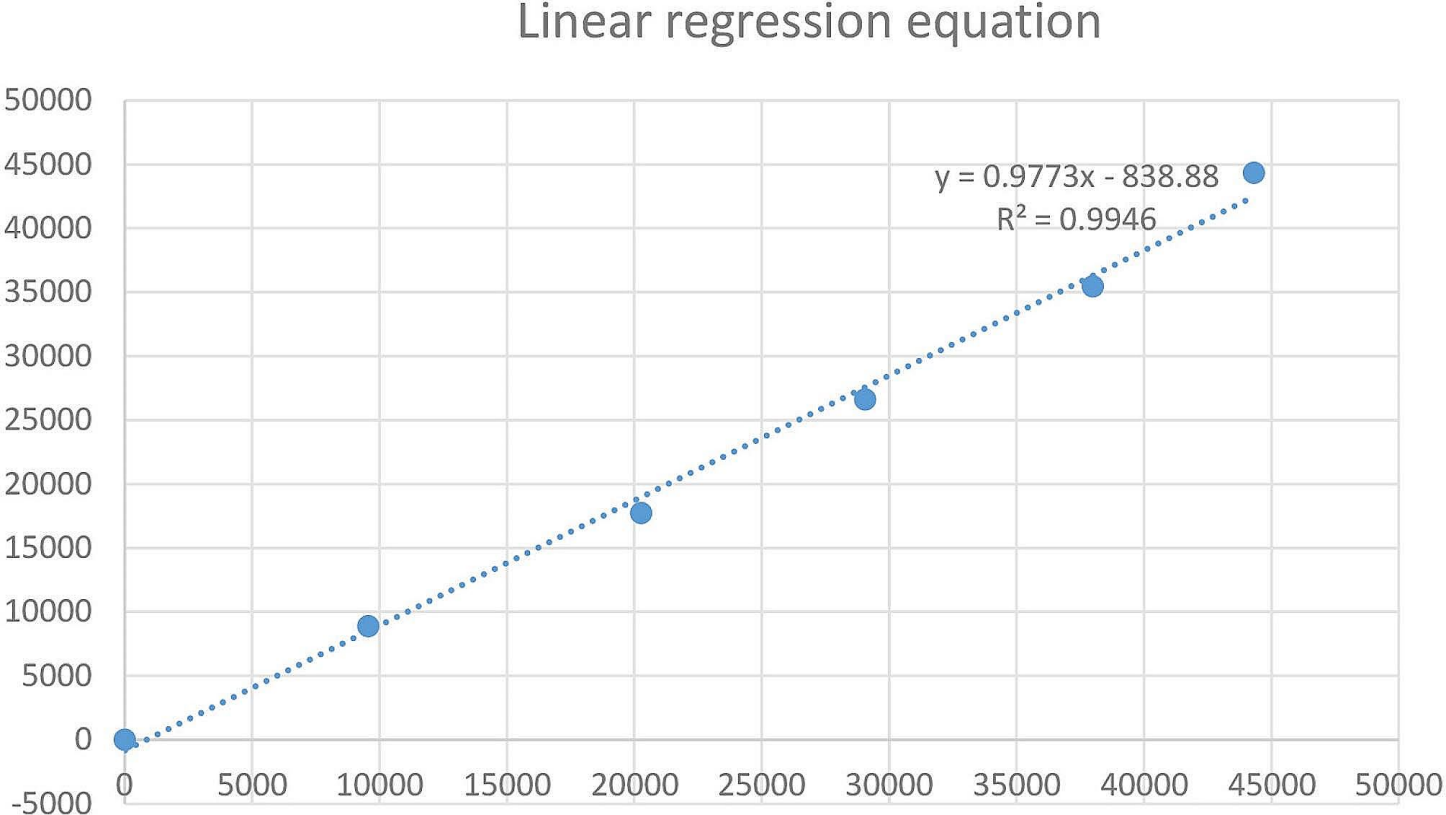
Linear regression equation of NT-proBNP

The linear range of NT-proBNP claimed by the manufacturer is 10–35,000 ng/L, and the linear range of verification is 7.2–44,313 ng/L. The linear regression equation is y = 0.9773x-838.88 with the slope of 0.9773, R^2^ = 0.9946, indicating that the detection reagent and the instrument have good linearity within the detection range claimed by the manufacturer **(**Fig. 4**)**. The linearity in low concentration interval (10 and 8,000 ng/L) was showed in supplementary Fig. 1.

### Detection limit

The LOB and LOD results of NT-proBNP are not higher than the manufacturer claims (LOB = 6 ng/L, LOD = 10ng/L, LOQ = 30 ng/L); The deviation of LOQ for all results is less than 30%.

### Method comparison

Serum samples from 724 heart failure patients (700 within the linearity range) were tested in CL-6000i and Cobas 601. The consistency regression equation between them is Y = 0.999x -1.177, R^2^ = 0.9890. The statistical coincidence rate of tested samples in 125ng/L and 300pg /mL were 99.3%, indicating good consistency **(**Table 4**)**.

**Table 4:** Agreement of NT-proBNP between cobas 601 and CL-6000i

	*125pg/mL*			*300pg/mL*				
	Roche positive	Roche Negative	Total		Roche positive	Roche Negative	Total	
Mindray Positive	496	1	497		408	5	413	
Mindray Negative	4	199	203		0	287	287	
Total	500	200	700		408	292	700	
Consistency Rate	99.2%	99.5%	99.3%		98.8%	100.0%	99.3%	

### Diagnostic performance

**Fig. 5:**
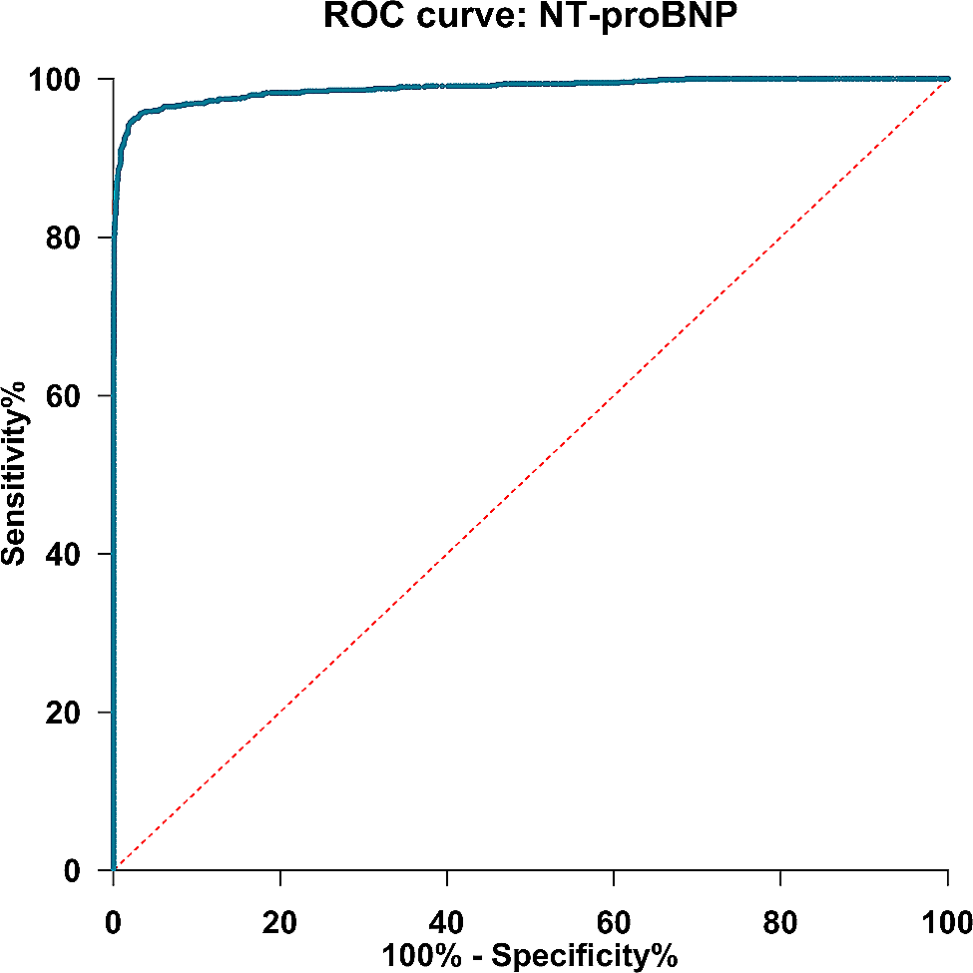
The ROC curve of NT-proBNP

Of the 970 CHF patients, there are 227 patients aged < 50 years old, 413 patients aged 50–75 years old, and 330 patients aged ≥ 75 years old. The value of NT-proBNP to diagnose CHF patients from healthy individuals was assessed with an ROC analysis **(**Fig. 5**)**. The area under the ROC curve was 0.9881 (95% CI: 0.9840 to 0.9922, *P* < 0.0001). The diagnostic performance of the cutoffs mentioned above is presented in Table 5.

**Table 5:** Diagnostic performance of NT-proBNP in cutoffs

Age	HF	Cutoffs (pg/mL)	Sensitivity	specificity	PPV	NPV
All age	Unlikely	125	96.60%	92.30%	84.20%	98.50%
All age		300	94.00%	98.20%	95.70%	97.50%
< 50 years old	Likely	450	89.40%	95.40%	77.80%	98.10%
50–75 years old		900	78.00%	98.30%	96.10%	89.20%
> 75 years old		1800	63.90%	99.60%	99.50%	67.20%

## Discussion

In our study, the distribution of NT-proBNP levels is influenced by age and gender. NT-proBNP increases significantly with age in both men and women, which is consistent with the results of many studies [15–18]. The mechanism behind this increase is not well understood, but it is suggested that BNP is affected by cGMP in the metabolic pathway, and its clearance rate decreases with age [18]. However, NT-proBNP does not bind with clearance receptors, and increased levels may result from decreased cardiac and renal function [19].

The study also found that NT-proBNP levels were lower in males than females, consistent with previous findings [19–21]. Suthahar N et al. [19] found that the level of NT-proBNP in women was nearly twice that in men, similar to our findings. The root cause of gender-related differences is still unclear, but related studies suggest that low haemoglobin in women or the influence of sex hormones may be the reasons. Estrogen is proven to upregulate natriuretic peptide expression, and Androgen mediates the difference. Further investigation is needed to understand the mechanisms affecting NT-proBNP levels [22–24].

Sex steroids and nitric oxide (NP) levels fluctuate throughout life, with high NP levels after birth and decreasing during the first months of life [25, 26]. High NP levels during neonatal periods may help the newborn’s cardiovascular system adapt to extra-uterine life conditions, alleviating increased ventricular afterload and supporting heart function [25, 27]. Adolescent girls have higher NP levels than boys, with fertile women showing two-fold higher BNP and NT-proBNP values than men [28]. Pregnancy is particularly affected, with early increases in BNP and NT-proBNP levels [29].

This research also found that when age is under 65 years old, the NT-proBNP level was significantly different between men and women (*P* < 0.05). When the age is no less than 65 years old, there was no statistical significance in the level between males and females (*P* > 0.05), indicating that the correlation between gender and NT-proBNP level gradually decreases in older healthy populations, which may caused by the influence differences of hormones in elder [22–24].

In terms of performance verification, precision, accuracy, linearity, detection limit, and method comparison all perform well. Precision is the performance analysis of random measurement error of a series of measurement results of uniform samples, reflecting the consistency of the results. An acceptable precision provides the premise and basis for other performance verification. Repeatability represents the variation produced under basically unchanged test conditions, while reproducibility considers the influence of operators, systems and other factors during the laboratory equipment operation. They represent the precision of two extreme conditions, usually presented by the coefficient of variation (CV). In this study, the repeatability and reproducibility precision of quality controls and serum samples near the given thresholds of NT-proBNP showed an acceptable performance, which is less than 1/4TEa and 1/3 TEa recognized by CLIA ‘88, respectively.

The study evaluated the accuracy, linearity, detection limit, method comparison, and coincidence rate verification of NT-proBNP samples. Accuracy was determined to be less than 1/2, with a smaller bias indicating higher accuracy. Linearity ensured consistency between measured and calculated values within the linear range, with linear regression parameters meeting requirements. The detection limit indicated the instrument’s sensitivity to small changes in concentration, with LOB, LOD, and LOQ meeting validation criteria. Method comparison and coincidence rate verification evaluated the comparability of test results between different instruments, considering factors such as specimen, reagent preparation, traceability, testing method, and process. The consistency and consistency were good between CL-6000i and Cobas 601 in testing NT-proBNP.

NT-proBNP is of substantial medical value in diagnosing suspected HF [6]. In this study, the mean under the area of the ROC curve for NT-proBNP is 0.9881, suggesting that NT-proBNP is a valuable marker in identifying CHF patients, which is similar in several studies [30–32]. In HF unlikely cutoffs recommended by ESC guidance, 125 ng/L is greater in sensitivity, and 300 ng/L is greater in specificity. In HF likely cutoffs in acute settings, the specificity is all greater than sensitivity, reflecting the high value of exclusion of NT-proBNP; the diagnostic performance of all cutoffs is similar in previous research [32, 33]. Also, the sample size of the sick and healthy populations is not equally correlated and should be considered.

Our study has some strength points related to the recommendations of the IFCC Committee on Clinical Applications of Cardiac Biomarkers; we used one immunoassay and avoided using different assays. We stratified data according to sex and age. Our study was in a special ethnic group that deserves consideration [34]. However, there are several limitations in the reference range in this study. The enrolled individuals are mainly residents of Guangzhou, Guangdong Province. There is no exact report on whether regional factors affect the concentration distribution of N levels fluctuate throughout life, with high NP levels after birth and decreasing during the first months of life T-proBNP. However, this reference range can not reflect the actual NT-proBNP levels of healthy adults in other regions. Also, half of the subjects were under 45 years old; HF is infrequent at this age, and the Body mass index was not included as a co-factor in this study. Therefore, we recommend conducting more comprehensive studies with a larger population to assess the effect of different demographic factors and comorbidities on the levels of NT-proBNP.

## Conclusions

The concentration of NT-proBNP levels was shown to be significantly affected by age and gender. Males had lower NT-proBNP levels than females. In older people, the relationship between gender and NT-proBNP level steadily weakens. The criteria were met in terms of performance verification, precision, accuracy, linearity, detection limit, and method comparability. We recommend adjusting cutoff values according to different demographics.

### Electronic supplementary material

Below is the link to the electronic supplementary material.

**Supplementary Material 1**: **Supplementary Table 1**. The data of CHF patients.

**Supplementary Material 2**: **Supplementary Fig. 1**. Linearity of NT-proBNP in Low concentration interval.

## Data Availability

The data used or analyzed during the current study are available from the corresponding author on reasonable request.
