# Supplementary figures and images for: Analytical and clinical performance evaluation of a new NT-proBNP assay
Source: BMC Cardiovasc Disord. 2024 Jul 5;24:341. doi: 10.1186/s12872-024-03994-w (PMC11229486; doi:10.1186/s12872-024-03994-w)

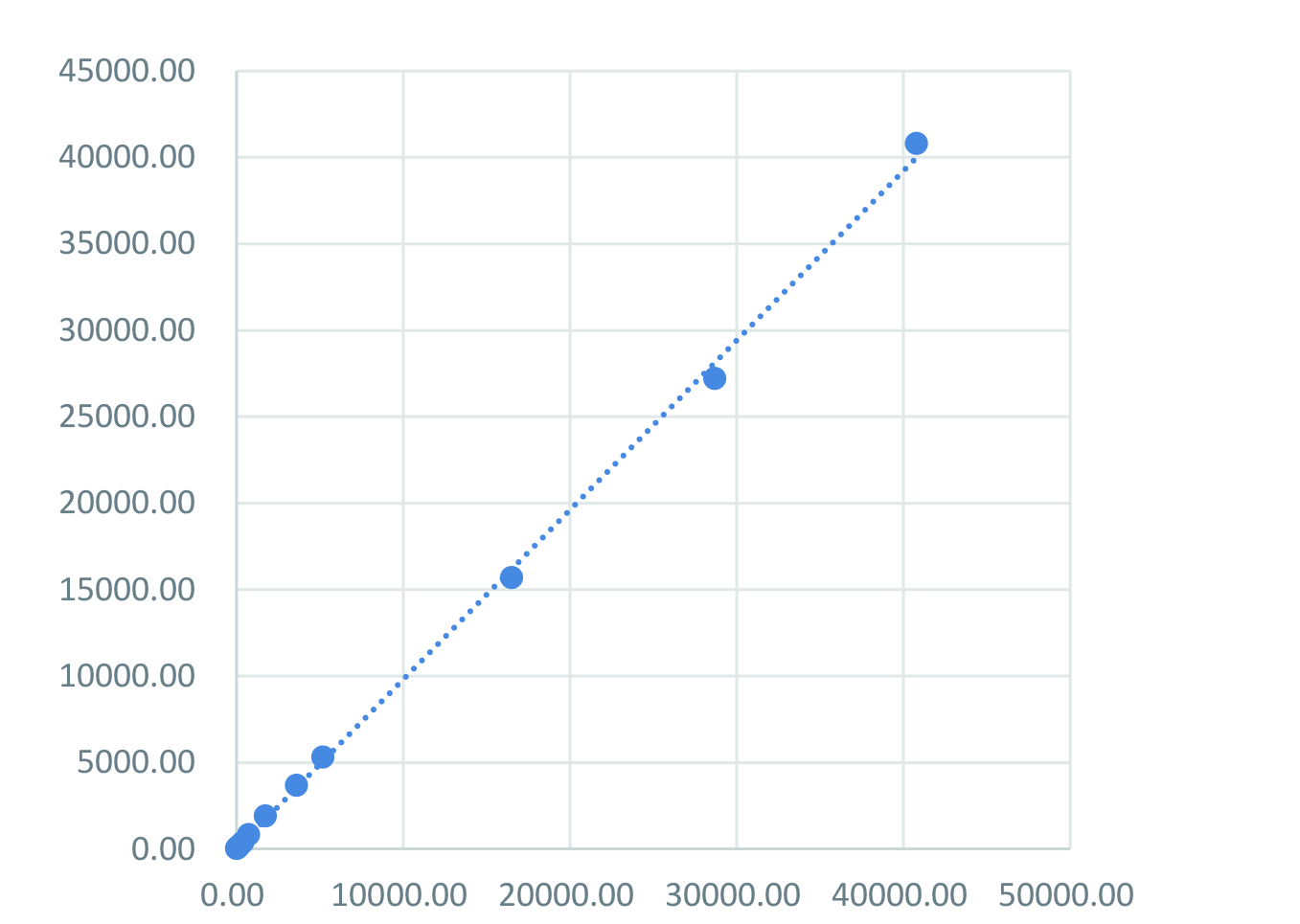


Supplementary figure 1. Linearity of NT-proBNP in Low concentration interval

Supplement: Supplementary file 2 — Supplementary Material 2: Supplementary Fig. 1. Linearity of NT-proBNP in Low concentration interval. [file 12872_2024_3994_MOESM2_ESM.docx]
